# Use of Voice Recordings in the Consultation of Patients Seeking Genital Gender-Affirming Surgery: An Opportunity for Broader Application Throughout Surgery?

**DOI:** 10.26502/jsr.10020269

**Published:** 2022-12-09

**Authors:** Shannon M Smith, Jenna Stelmar, Grace Lee, Peter R Carroll, Maurice M Garcia

**Affiliations:** 1 Cedars-Sinai Medical Center, Los Angeles, Division of Urology; Los Angeles, CA, USA; 2 Cedars-Sinai Transgender Surgery and Health Program; Los Angeles, CA, USA; 3 University of California San Francisco; Department of Urology; San Francisco, CA, USA; 4 University of California San Francisco; Department of Anatomy; San Francisco, CA, USA

**Keywords:** Patient Satisfaction, Sound Recording, Physician-Patient Relations, Decision Making, Empowerment

## Abstract

**Introduction:**

It has been demonstrated that patient memory for medical information is often poor and inaccurate. The use of audio recordings for patient consultation has been described; however, to our knowledge this is the first reported use of audio recordings in consultation for gender-affirming surgery. Our aim was to determine whether, and specifically how, audio recording the consultation of patients presenting for genital gender-affirming surgery would be of benefit to patients.

**Materials and Methods:**

We began to offer all new patients the opportunity to have their consultations recorded. At the end of the consultation the recording was uploaded to a USB, which was given to the patient to keep. We then surveyed all patients who had received a copy of their recorded consultation to query the utility of having access to an audio recording of their consultation.

**Results:**

71/72 (98.6%) patients who were given the option to have their consultation recorded chose to do so. 50/71 (70%) of patients who had their consultation recorded responded to our survey. Patients reported that having access to a voice recording of their consultation was beneficial and was viewed overwhelmingly positively.

**Conclusions:**

Routine audio recording of patient consultations is highly beneficial to patients, with little cost to providers, and should be considered as a valuable addition to the new patient consultation. This approach may have applications in broader clinical contexts where patients face numerous, complex, and nuanced management options. The study would benefit from continued application and a larger (multi-center, international) sample.

## Introduction

Patient recall of medical information discussed in the outpatient clinic setting can be limited and inaccurate [1–4]. Proposed interventions to help improve patient recall of information shared during consultation include: providing patients with a summary letter [5], question prompt sheets [6], audio recording the consultation [7–11], or video recording the consultation [12,13]. The use of consultation audio recordings has primarily been investigated in the setting of oncologic consultation [14–20], but has been described in other patient settings as well [4,8,12,13,21] including a randomized controlled trial in over 4300 patients in a multispecialty setting [21]. We sought to answer the question “Would audio recording the consultation of patients presenting for gender-affirming surgery be of benefit to the patients, and if so, how?”

At our center, a new patient consultation for gender-affirming surgery is scheduled for 60 minutes. Our new patient visit provides for a comprehensive overview about all surgical options that we offer (and those we do not) and are presented together with the pros and cons of each option. We include focused discussion about the ways in which some options can either facilitate or preclude other surgical options or features. During these lengthy new patient discussions, the large volume of information that is shared with patients can be overwhelming and difficult for them to remember.

We hypothesized that patients presenting to our clinic for new patient consultation to discuss surgery options would benefit if provided with a voice recording of their consultation. To our knowledge, this is the first study examining the use of voice recordings in the surgical consultation process for patients seeking genital gender-affirming surgery.

## Materials and Methods

Between April 2021 and July 2022, we offered all new patients the option to have their initial consultation with their surgeon recorded using a hand-held digital Dictaphone (Sony, New York, USA), and to be provided with a copy of the voice recording on a USB flash drive, all at no cost to the patient. The study protocol was reviewed and approved by our institution’s clinical research Institutional Review Board (IRB Approval #00001698). We consulted our institution’s Risk Management Office, which informed us that California law requires two-party consent for either party to make an audio recording of the other. Therefore, we would have to document the patient’s consent to be recorded in the patient’s medical record, and that we should keep a copy of the recording as well.

At the commencement of the recording, the surgeon dictated the patient’s full name and a statement that they give permission for the visit to be recorded. At the end of the consultation, an audio file containing the recording was uploaded to a USB, which was either immediately given to the patient or was mailed to their home address within 1–2 days of their consultation.

At least two weeks after each patient’s consultation, we sent an email with a link to a questionnaire featured on an internet-based software platform designed to completely preserve patient’s anonymity (Qualtrics, Utah, USA). Our intent was to query whether, and how, the recording may (or may not) have been useful to them. Within the questionnaire, we provided free-text space for patients to share their overall feedback, other details about how the patient used the recorded copy provided to them, and any other comments they wished to provide.

During the study we decided to include a validated questionnaire, the Shared Decision-Making Questionnaire (SDM-Q-9) [22]. We also decided to ask patients 3 additional questions querying what proportion of the entire recording they listened to and how soon after receiving the recording they first listened to it, and what percentage of the original consultation discussion they believe they would have remembered and not remembered without being able to listen to the audio recording. Because the questionnaires were provided to subjects at a specific time after their consultation, the initial 24/50 patients were not presented with these additional questions, but the most recent 26/50 patients did complete these questions.

With respect to surgery decision making, in our practice we emphasize to every patient the responsibilities we as providers have, and what the patient’s ultimate responsibility is. We explain that our role as providers is to provide information about all existing surgery options and the attendant risks and potential benefits of each to them, so that the patient can make the most informed decision possible about what surgery option best aligns with their personal priorities. We emphasize that ultimately the decision for or against any particular surgery is completely and exclusively theirs to make.

## Results

**Figure 1:**
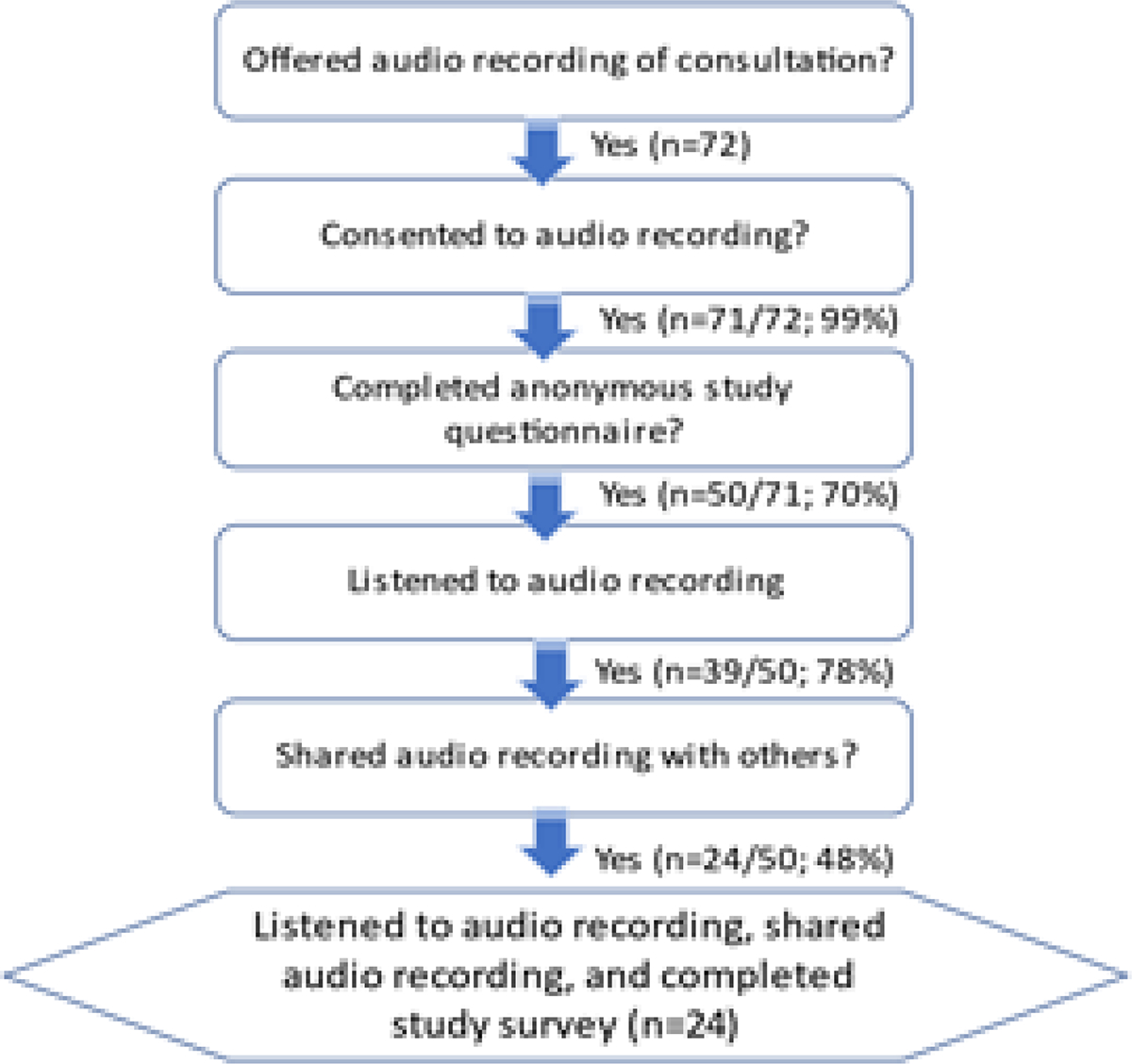
Flowchart of patients who participated in the study. Of the 71 patients who received the questionnaire, 50 responded to our survey. Of note, some of the questions were not answered by all 50 patients, so some of the questions have slightly different numbers of respondents.

A total of 71/72 (98.6%) consecutive patients who were given the option to have their consultation recorded chose to do so (Figure 1). The one person who declined stated their reason for declining was that they did not believe they would use it. Of the 71 patients who received the questionnaire, 50 responded to our survey (20 transfeminine, 25 transmasculine, 2 gender non-binary, and 3 cisgender men). Mean age (±SD) was 35.4 (±14.8) years. Of note, some of the questions were not answered by all 50 patients, so some of the questions have slightly different numbers of respondents.

**Figure 2:**
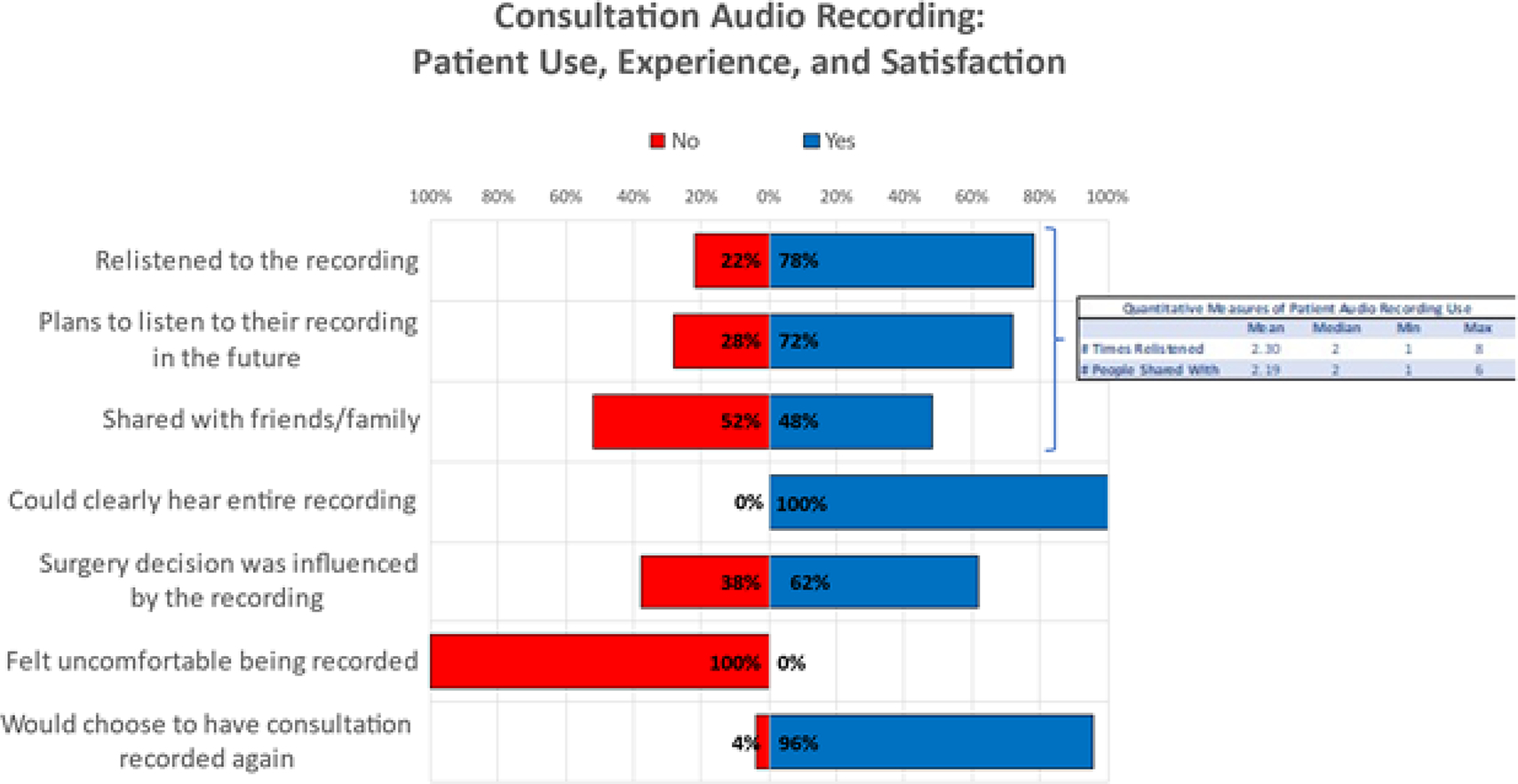
Qualitative and quantitative measures of patient experience with the audio recording

Twenty-four out of fifty (48.0%) of patients reported that they shared their recording with family and/or friends who could not attend the consultation (Figure 2). The mean number of people with whom they shared the recording was 2.19 (median=2, range= 1–6). When asked who they shared their recording with, patients primarily indicated their significant other(s) (38%) and their friends (35%). Thirty-nine out of fifty (78.0%) of patients reported that they listened to their recording after their consultation. The mean number of times listened was 2.30 (median=2, range 1–8).

When patients were asked whether at any point they felt uncomfortable about having their consultation recorded, 100% of respondents reported “no.” When patients were asked “If you had your initial consultation all over again, would you choose to have your consultation recorded and shared with you?” 48/50 (96.0%) responded “yes.” Two patients responded “no,” with one patient using the free-text box provided to elaborate as to *why* they would not opt for the recording again. This patient stated that they were presenting for an orchiectomy, a “simple procedure with few complications” that they had “already thoroughly research beforehand,” but if they were having a more complicated gender-affirming surgery, they would have found the recording helpful.

The specific portions of the recorded visit that patients reported listening to most frequently (detailed here in ranked order) concerned: #1. Detailed descriptions of the different surgical options; #2. The potential risks associated with the surgeries discussed; and #3. The potential benefits associated with the surgeries discussed.

**Figure 3:**
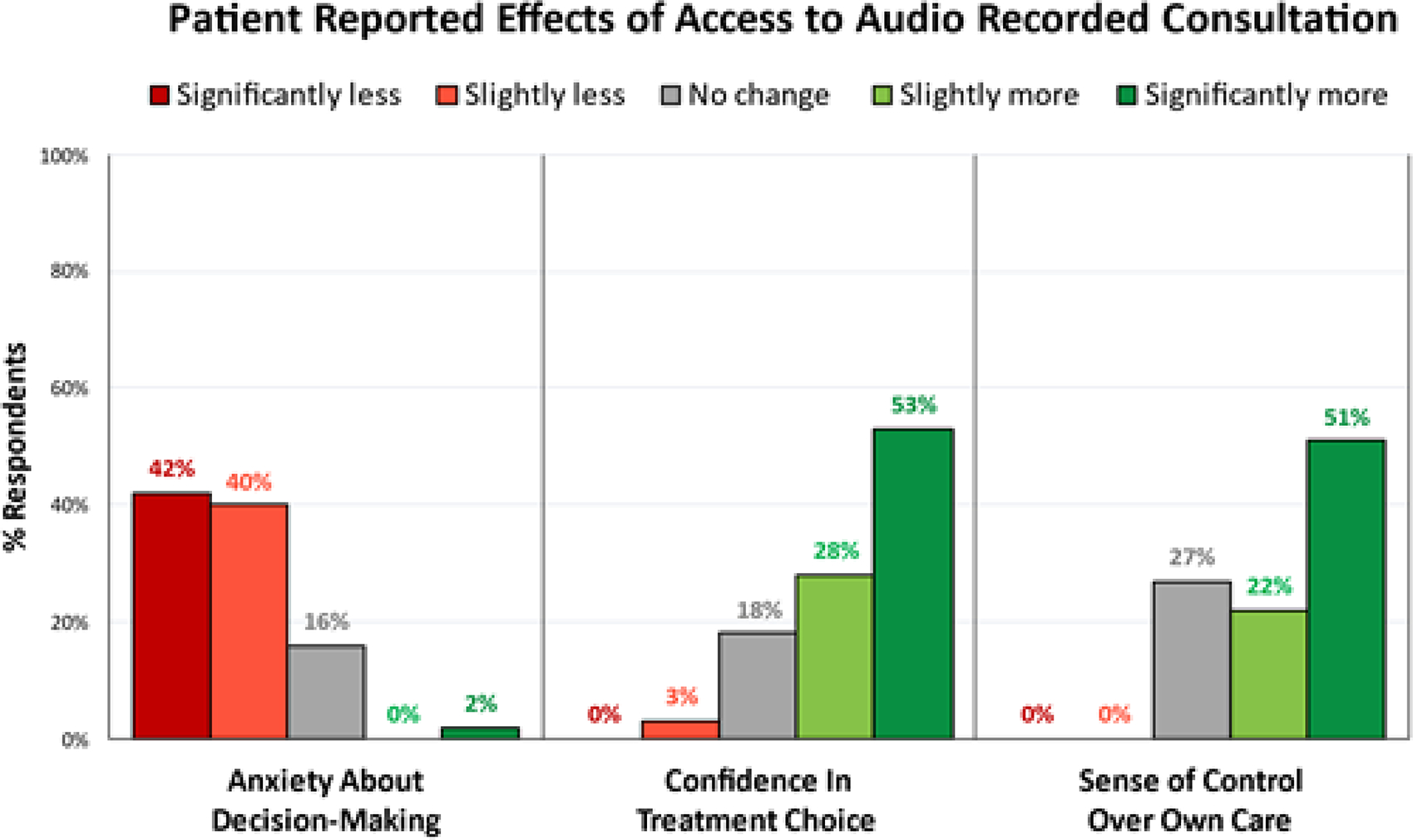
Patients reported that having access to their recorded consultation lessened their anxiety about decision making, increased their confidence in their treatment choice, and increased their sense of control over their own care.

When patients were asked whether *listening* to the recording changed how confident they felt about the choice for surgery, 39/40 (97%) of subjects’ responses were either neutral or positive, (7 reported “no change,” 11 reported feeling “slightly more confident,” and 21 reported feeling “significantly more confident”) (Figure 3). When asked whether they found it helpful to have a recording of the consultation *available,* 100% of the 40 respondents answered in the neutral or affirmative: 1 patient answered “neutral,” 5 patients answered “probably yes,” and 34 answered “definitely yes.”

When patients were asked whether *having access to* the recording (regardless of whether they listened to it or not) affected their sense of control over their treatment, all 45 responses were either neutral or positive, (12 reported no effect, 10 slightly more in control, 23 significantly more in control) (Figure 3). When asked whether having access to a recorded copy of their consultation affected any anxiety they had about choosing their treatment, 19/45 (42%) of patients reported it made them feel “significantly less anxious,” 18/45 (40%) “slightly less anxious,” 7/45 (16%) “neutral,” and 1/45 (2%) “significantly more anxious.”

**Figure 4:**
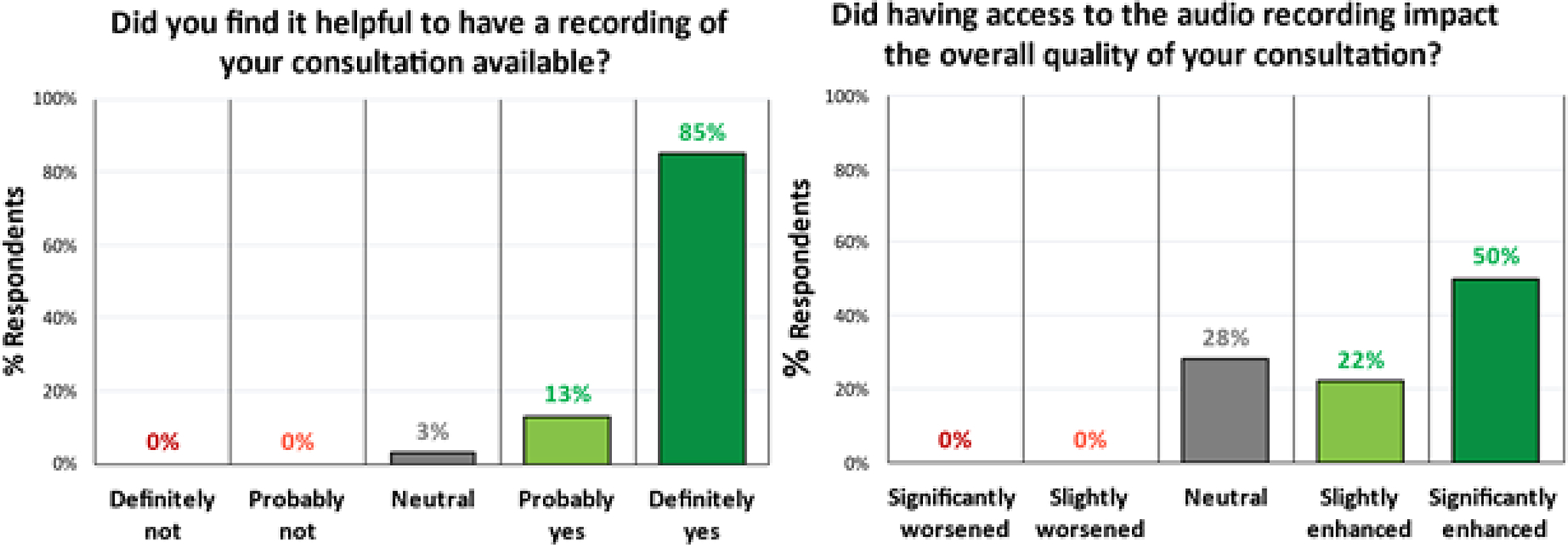
Patients reported they found it helpful to have the recording available, and also reported that it enhanced the overall quality of their consultation.

When subjects were asked whether the recording increased their subjective sense of overall quality of the consultation, all 50 who responded to this question were either neutral or positive: 14 (28%) reported “no impact”, 11 (22%) reported that the audio recording “slightly enhanced” their perceived quality of the consultation, and 25 (50%) reported the audio recording “significantly enhanced” their perceived quality of the consultation (Figure 4).

**Figure 5a.**
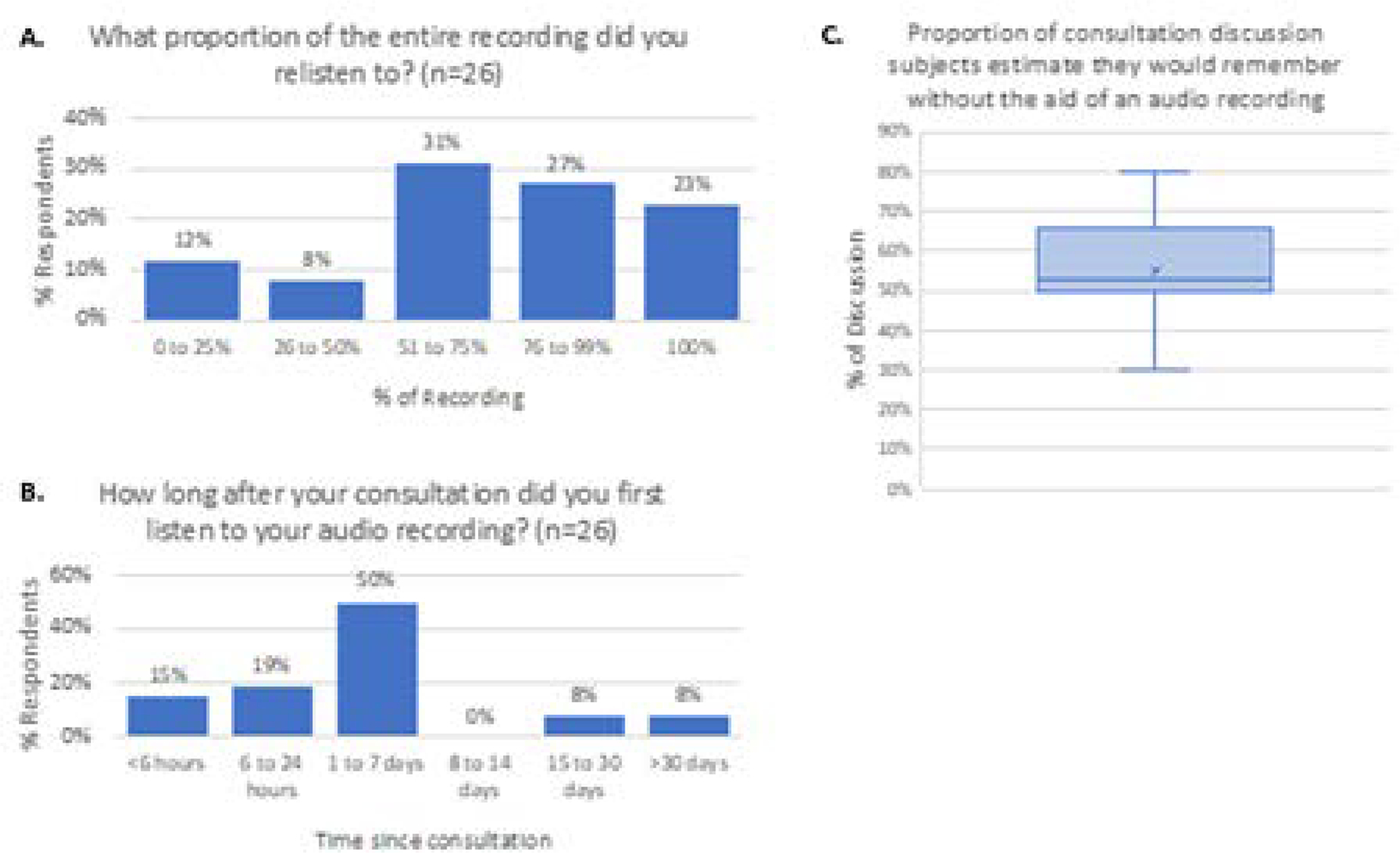
Percent of the total recording that patients listened to. **5b.** Time from initial consultation to listening to the recording. **5c.** Estimated proportion of consultation the patients would have remembered without access to the audio recording. Horizontal line represents the median, x represents the mean.

**Figure 6:**
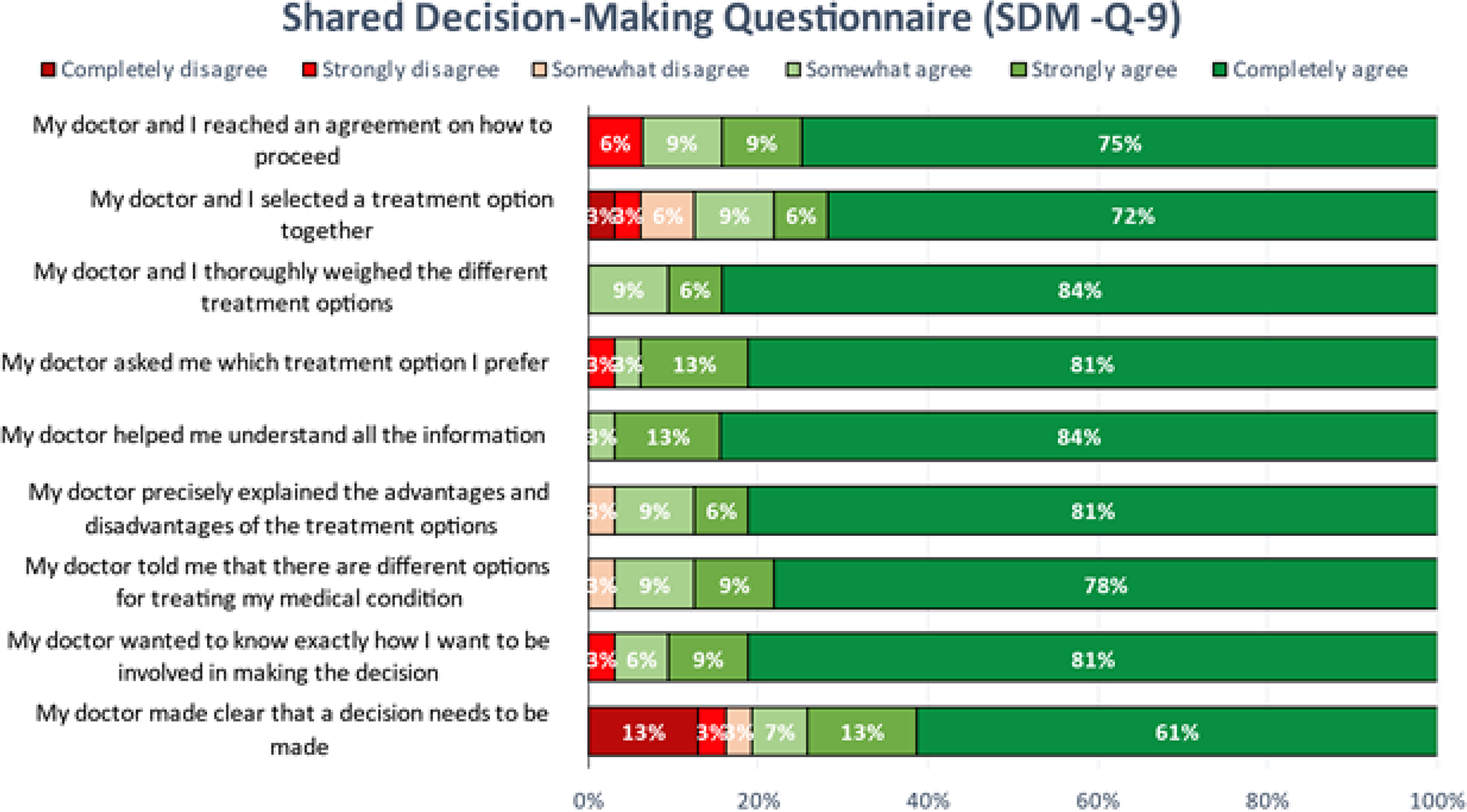
Results of the Shared Decision Making Questionnaire (SDMQ-9).

The most recent 26/50 (52%) patients in our series responded to the 3 additional questions and Shared Decision-Making Questionnaire (SDM-Q-9) as shown in figure 5 & figure 6, respectively. Eighty percent of this patient cohort re-listened to ≥51% of the audio-recording. Thirty-four percent of these same patients listened to the audio-recording for the first time within 24 hours of their consultation, and 84% listened to it within 7 days (Figure 5A & 5B). This cohort of patients estimated that, without the aid of the audio recording they would have remembered only a mean of 53% of the information from the consultation (Figure 5C). The results of the *Shared Decision-Making Questionnaire (SDM-Q-9)* are shown in figure 6.

## Discussion

### Potential Benefits

Based on this initial work, the feedback that we have received from patients has been overwhelmingly positive, and in favor of recording the consultations and then providing the voice recording to patients. It is interesting to note that while only 78% of patients listened to the recording again, and only 48% of patients chose to share it with friends and family, almost all patients who responded to the survey reported that simply *having access to* the recording positively affected their sense of control, lessened their anxiety about their care, and had a positive impact on their perceived overall quality of the consultation. This is consistent with findings from previous studies which have shown a reduction in anxiety, better informed decision making, and improved confidence in their treatment decisions [23]. Patients report feeling more empowered by having unrestricted access to a voice recording [24,25]. Many patients also find voice recordings to be an opportunity to improve their own care [26]. Many also report that audio recordings improve their relationship with their health professional [21].

Patients have indicated desire to replay, relisten, and to share their consultation recording with others [25]. Indeed, 80% of patients in one of our patient cohorts listened to more than half of the recording, and 30% listened to the recording within 24 hours of their consultation (Figure 5). Audio recording allows patients to control who they share their consultation with, and when. This is especially important in the present era of the COVID pandemic, when friends and family members are often excluded from attending in office visits to reduce physician/patient risk of exposure to COVID. Consequently, many patients do not have the benefit of a ‘second set of ears’ from accompanying friends and family to help the patient process all the information they receive during such consultations. Having the recording allows the patient to share with friends and family who might not be able to accompany the patient at their appointment, so that the latter may participate in the patient’s care.

An audio-recording of a consultation also captures nuances of the provider’s discussion that even a written transcript (or an accompanying friend/family) cannot relay in the same fashion after the visit. Examples include a tone (serious, concerned, confident, skeptical), empathy, sincerity, and honesty. Other examples include the opportunity to define what exactly is meant by “shared decision-making” to a particular provider. In our practice, the only “shared” aspect of decision-making we (as providers) assume responsibility for is explaining what surgical options are simply not possible due to technical impossibility or exceedingly high safety-risks. Short of these, all decisions are made by the patient. Other surgeons (and surgical specialties) may claim more of a role in decision-making. Clearly, “shared decision making” may have a different definition by individual surgeon and sub-specialty. Regardless, all patients are entitled to transparency about decision-making roles during care.

Because audio recordings do capture tine and all details of a discussion, their use also demands that providers make an effort to avoid conveying a dismissive tone or lack of respect to patients, and that their discussion truly be patient-centered and comprehensive. Validated questionnaires about decision-making should query whether or not a provider attempted to define their and the patient’s decision-making roles.

### Potential barriers and concerns to routine utilization of consultation audio-recordings

A significant barrier to widespread utilization of routinely audio recording consultations is provider concern about potential downsides and/or risks [9,10,24,26,27]. Some of the concerns providers have shared include that routinely recording consultations could be burdensome or disruptive to the clinic [26], concern for the availability of funding for such a program [26], and potential medicolegal concerns as a barrier to implementation of routine consultation audio recording [8–10,27].

#### Cost:

There is a fixed cost associated with providing the voice recordings to patients. First, there is the one-time expense of the recording device. The new, digital recording Dictaphone device we purchased (Sony, New York, USA) was $42.66 after tax. We purchase the USBs we used in packages of 10 units that cost $32.70, for an individual cost of $3.27 per USB per patient. While this is an additional expense, we believe that this expense is minimal, especially in comparison to the benefit obtained for the patients.

#### Medicolegal concerns:

Some providers have expressed concern that patients could potentially use the recording against them in court should the patient file a lawsuit against the provider [8–10,27]. We take the position that any conversation with a patient should always provide the complete, true, and correct information that the provider intends to provide. A provider’s knowledge that the conversation is being recorded should not affect what the provider says to the patient during the consultation. If this standard of transparency is adhered to during all patient consultations, access to the recorded consultation by the patient or their attorney should, if anything, only serve to *protect* the provider. Regarding legal risk, it should be noted that in a large study of over 2800 patients who had their consultations recorded, none (0%) of those patients had used their recorded consultations as part of a medicolegal action [12].

A potential concern with use of audio-recordings is that the patient could, in theory, attempt to manipulate it in a way that distorts what the provider said, in such a way that favors the patient in a lawsuit. This theoretical risk is unlikely if only because forensic methods are likely to detect post-hoc manipulation of a recording and addition of voice-over recordings. Furthermore, this risk is entirely eliminated when the provider keeps a copy of the audio-recording for their own records (as we did in our study) for use, when needed, to compare to a version of the audio-recording suspected of having been manipulated.

#### Consent to audio-record:

It is important to be aware of the state and federal laws that govern the recording of conversations between parties. Federal law requires one-party consent, which means one can record a phone call or conversation so long as they are a party to the conversation. If they are not a party to the conversation, a conversation or phone call can be recorded only if at least one party consents to recording and has full knowledge that the communication will be recorded. Thirty-five states and the District of Columbia follow the federal law and require only one-party consent, while fifteen states, including our state (California) require all-party consent to record a conversation [28]. For this reason we take care to ensure that the patient’s consent to voice recording is well-documented. Immediately after commencement of the recording we read the patient’s name aloud and state that the patient agrees to the consultation being recorded. We also document in the written medical record that the patient has elected to have the consultation recorded so that they may retain a copy for their potential benefit. Lastly, at the recommendation of our Office of Risk Management, we retain a copy of any recording we give to patients and enter this into their confidential medical records.

### Alternatives to voice recording

It is worthwhile to consider alternative options to audio recordings. With the growing utilization of telemedicine, many patient consultations take place by Zoom^®^, Microsoft Teams^®^, and similar platforms. A potential limitation of video-based platforms is that telemedicine visits include video of both physician and patient, some patients might be reticent about sharing a copy of the video-recorded discussion that includes the patient in view. By eliminating the patient from “view”, a voice recording centers the focus on the information that the physician is sharing. Another potential limitation is that if a video recording were shared by a contact of the patient without authorization by the patient, the presence of the patient in the video poses a greater risk to loss of privacy as compared to an audio-only recording. Nonetheless, some patients could certainly find sharing a telemedicine video recording a useful alternative.

When given the option to choose between audio recording and other aid strategies such as letters or transcriptions of the consultation to enhance information recall, patients prefer audio recordings. [10] As providers, we prefer providing patients with a voice recording versus a transcription of the encounter, for several reasons. 1) Nuances from conversation (tone, emphasis) are preserved, as compared to being lost with transcription. 2) Transcription of a conversation requires additional time, resources, and potential loss of confidentiality; 3) With transcription, there is a risk that the conversation may be transcribed incorrectly (by a person or a machine) and require proof-reading by a human or by artificial intelligence software. Also, the provider is ultimately responsible (and accountable) for the transcribed content they provide to patients.

## Conclusion

This study contributes to a growing body of work that demonstrates the utility and benefit of audio recording patient consultations. This appears to be especially useful with complex consultations such as our gGAS new patient consultations. With a voice recording, patients are able to re-listen to the discussion of the available options, to better understand and absorb the discussion, and, if they decide to share their recording with others, they have the option of obtaining input about the surgery options they are considering.

Future work should include randomized trials to compare these metrics between those who did and did not have their consultations recorded. Future studies should also include other surgical clinical contexts where patients face numerous, complex, and nuanced management options. Examples include a patient newly diagnosed with prostate cancer who must decide between active surveillance, surgery, radiotherapy, or focal therapy. For clinical scenarios where an abundance of options, competing risk data, widely varying clinical courses, and anxiety may overwhelm patient understanding and recall, the opportunity to review the discussion at home or with loved ones can improve decision-making. Future work should also critically assess how well we do in communicating choices and risk to patients.

As more patient-centered care continues to evolve, in addition to offering innovative treatments for patients to choose from, it is important that we strive to also provide patients with tools, such as the in-office consultation voice recording approach we describe, to help them feel better informed and more empowered and confident in their own healthcare decisions. Our group has developed a smartphone App (Visit Replay^©^; U.S. and International Patents Pending) that creates and archives office-consultation audio-recordings (which can also be transcribed, if desired), and includes a platform to communicate with patients to better understand how they value the different elements of the consultation and to enhance their knowledge about their treatment options. Future work will assess the efficacy of this App and its utility across specialties outside of urology.
